# Near‐Infrared Persistent Luminescence Nanoprobe for Ultrasensitive Image‐Guided Tumor Resection

**DOI:** 10.1002/advs.202207486

**Published:** 2023-04-23

**Authors:** Peng Lin, Junpeng Shi, Ye Lin, Qian Zhang, Kexin Yu, Lin Liu, Liang Song, Yile Kang, Maochun Hong, Yun Zhang

**Affiliations:** ^1^ School of Rare Earths University of Science and Technology of China Hefei 230026 China; ^2^ Ganjiang Innovation Academy Chinese Academy of Science Ganzhou 341000 China; ^3^ State Key Laboratory of Structural Chemistry Fujian Institute of Research on the Structure of Matter Chinese Academy of Sciences Fuzhou 350002 China; ^4^ Xiamen Key Laboratory of Rare Earth Photoelectric Functional Materials Xiamen Institute of Rare Earth Materials Haixi Institute Chinese Academy of Sciences Fuzhou 350002 China

**Keywords:** image‐guided surgery, manganese dioxide, persistent luminescence nanoparticles, tumor residues recognition, ultrasensitive nanoprobe

## Abstract

Near‐infrared (NIR) fluorescence imaging poses significant superiority over traditional medical imaging for tumor resection, thus having attracted widely attention. However, for tiny tumor residues, it requires relative high sensitivity to determine. Here, based on persistent luminescence nanoparticles (PLNPs), an ultrasensitive nanoprobe with extraordinary tumor imaging result is developed to guide surgical removal. Persistent luminescence (PersL) is quenched in normal tissue by the outer layer of MnO_2_, and is recovered due to the degradation of MnO_2_ in tumor microenvironment, significantly improving the sensitivity of tumor imaging. Combined with the absence of background fluorescence in imaging of PLNPs, ultrahigh sensitivity is achieved. In orthotopic breast cancer model, the intraoperative tumor‐to‐normal tissue (T/NT) signal ratio of the nanoprobe is 58.8, about 9 times that of downconversion nanoparticles. The T/NT ratio of residual tumor (<2 mm) remains 12.4, considerably high to distinguish tumor tissue from normal tissue. Besides, multiple‐microtumor, 4T1 liver‐implanted tumor and lung metastasis models are built to prove that this ultrasensitive nanoprobe is feasible to recognize tumor residues. Notably, PersL imaging takes only 1.5 min, appropriate to be applied for intraoperative imaging. Overall, an ultrasensitive and convenient imaging for recognizing residual tumor tissue is introduced, holding promise for complete surgical removal.

## Graphical abstract

An intelligently responsive nanoprobe with ultrahigh sensitivity for image‐guided tumor resection is developed. Based on persistent luminescence (PersL) imaging without autofluorescence, signal is quenched by the outer layer of MnO_2_ in normal tissue, and recover in tumor region due to the degradation of MnO_2_. It provides ultrahigh tumor‐to‐normal tissue (T/NT) signal ratio to identify tumor residues during surgical removal.

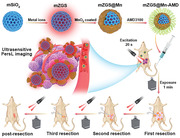

## 1 Introduction

Cancer seriously threats human health due to lack of effective treatment. Currently, surgical removal is still a preferred treatment for solid tumor and is sometimes the only performable option.^[^ ^1^ ^]^ After surgery, the residual tumor tissues often cause to local cancer recurrence and deterioration that indicates failure of operation. Under the premise of protecting the normal tissue, how to completely remove the tumor is the challenge of surgery. Preoperative medical imaging technology such as MRI, CT, or X‐ray are used to estimate the position and size of tumor. During surgery, surgeons rely on visual, touch, and experience to distinguish tumor margin from healthy tissues, often causing leaving tumor residues or removing normal tissues excessively.^[^ ^2^ ^]^ Without intraoperative real‐time navigation, this pattern restricts the precision of tumor resection and leads to an 8–70% probability of tumor residues.^[^ ^3^ ^]^

Compared with traditional medical imaging, near‐infrared (NIR) fluorescence imaging has the advantage of real‐time, high sensitivity, high resolution, no radiation, etc.^[^ ^4^ ^]^ Intraoperative NIR fluorescence imaging provides real time tumor location and its borderline to navigate for accurate surgical removal. Nowadays tumor resection assisted by NIR fluorescence imaging has attracted a number of researchers.^[^ ^5^ ^]^ The NIR fluorophore indocyanine green (ICG) has been approved for clinical use by the US Food and Drug Administration (FDA).^[^ ^6^ ^]^ However, the poor stability, photobleaching, and lack of tumor targeting of ICG limit the application of ICG in accurate tumor resection. With the improvement of nanotechnology, various NIR fluorescence nanoprobes have been developed such as quantum dot (QD),^[^ ^7^ ^]^ aggregation‐induced emission (AIE) nanoparticles,^[^ ^8^ ^]^ downconversion nanoparticles (DCNPs),^[^ ^9^ ^]^ etc. Because of the advantages of good photostability, tunable spectrum, and easy surface modification, NIR fluorescence nanoprobes are excellent tracers in tumor resection, and have been widely applied in related research.^[^ ^10^ ^]^ During surgery, as resection goes on, the remaining tumor get smaller and smaller, requiring more sensitive imaging nanoprobes. Nevertheless, during excitation of above‐mentioned nanoprobes, organisms emit autofluorescence, resulting in strong background signal and low tumor‐to‐normal tissue (T/NT) signal ratio, restricting tumor residues to be identified.

NIR persistent luminescence nanoparticles (PLNPs), which store energy during excitation and continue to emit afterglow after excitation, are a new kind of bioimaging nanoprobe.^[^ ^11^ ^]^ Using the NIR persistent luminescence (PersL) for bioimaging could completely avoid the interference of autofluorescence caused by the excitation irradiation, significantly improving the imaging sensitivity.^[^ ^12^ ^]^ In comparison with that of organic dye and QD, the emission signal of PLNPs without autofluorescence interference could clearly distinguish tumor from normal tissues.^[^ ^13^ ^]^ This feature of PLNPs, which provides high T/NT ratio, is suitable for the utility in imaging to guide tumor resection. However, comprehensive and insightful studies on PLNPs for image‐guided tumor resection have been rarely reported so far.

Here, we report an intelligently responsive PLNPs nanoprobe with ultrahigh sensitivity for image‐guided tumor resection in various tumor models. The ultrasensitive imaging affords considerably high T/NT ratio, allowing to clearly distinguish the residual tumor tissues. The MnO_2_ shell, grown outside the PLNPs, quenched the PersL in normal tissues, and was degraded in tumor microenvironment (TME) thanks to the excess H_2_O_2_ and glutathione, resulting in PersL recovery.^[^ ^14^ ^]^ This process of PersL improvement in tumor region leads to the PersL signal at tumor site much higher than normal tissues, which means higher sensitivity of this nanoprobe that that of pure PLNPs. Notably, whole process of imaging took less than 1.5 min, which indicates it is convenient for intraoperative application. During orthotopic breast tumor resection, the ultrahigh T/NT ratio of 58.8 was obtained and the T/NT ratio of <2 mm residual tumor tissue was 12.4. In multiple microtumor model and 4T1 liver‐implanted tumor model, the nanoprobe afforded significantly high intraoperative T/NT signal ratio of tumor residues as well. Besides, the highly sensitive imaging is promising to guide metastatic tumor resection.

## 2 Results and Discussion

### 2.1 Synthesis and Characterization of mZGS@Mn‐AMD

**Scheme 1:**
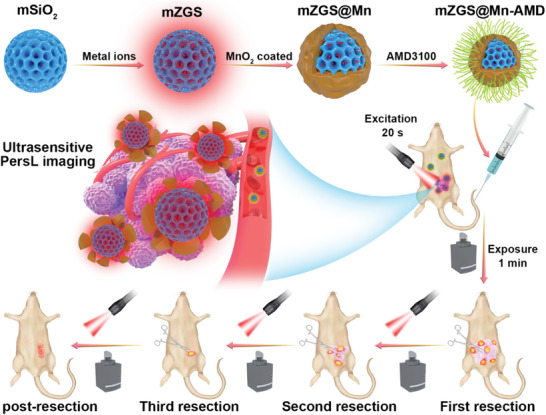
Schematic illustrating mZGS@Mn‐AMD fabrication for ultrasensitive PersL image‐guided tumor resection.

**Figure 1:**
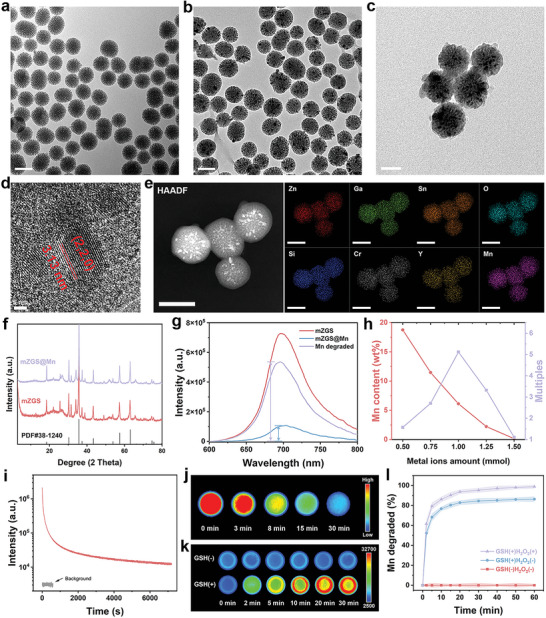
Characterization of samples. TEM images of a) mSiO_2_, b) mZGS, and c) mZGS@Mn. d) HRTEM image and e) element mapping images of mZGS@Mn. f) XRD patterns. g) PersL spectra. h) Relationship between metal ion addition amount and manganese content, afterglow enhancement times. i) PersL decay spectrum. j) PersL decay images of mZGS. k) PersL images of mZGS@Mn with or without GSH. l) Accumulated release profile of MnO_2_ under different conditions (Concentration of GSH = 0.01 m, concentration of H_2_O_2_ = 5 × 10^−5^ m, *n* = 3). All the tests related to PersL were carried out after irradiated with red LED for 20 s (wavelength peaking at 659 nm, 40 mW cm^−2^).

Zn_1.3_Ga_1.4_Sn_0.3_O_4_:Cr_0.005_, Y_0.003_ (ZGS) emits stronger NIR PersL after biological window red light excitation, demonstrated by our previous research.^[^ ^15^ ^]^ Due to the advantage of ZGS for bioimaging, it was selected as the core of nanoprobe. The procedure for synthesis of the nanoprobe based on ZGS was shown in **Scheme** **1**. The synthesis method of ZGS was silica template method, through which the thickness of MnO_2_ shell was allowed to be controlled by adjusting the addition amount of metal ions. MnO_2_ shell was directly grown outside the mSiO_2_ to obtain mZGS@MnO_2_ (mZGS@Mn). After surface modification of mZGS@Mn, AMD3100, which is a recognized CXCR4 antagonist and has been approved by FDA, was chosen as 4T1 cells targeting molecule to conjugated with mZGS@Mn by amidation reaction to prepare mZGS@Mn‐AMD.^[^ ^16^ ^]^ The uniform monodispersed mesoporous SiO_2_ (mSiO_2_) was successfully synthesized as shown in **Figure** **1**a. The uniform pore structure was also observed through transmission electron microscope (TEM) images. mZGS kept monodispersed with dynamic diameter of 121.2 ± 10.8 nm and the super small size darker nanoparticles can be observed among the mSiO_2_ due to the higher atomic number of ZGS (Figure 1b). The pore structure can still be observed, allowing MnO_2_ to grow in situ. mZGS was coated with an irregular shell as shown in Figure 1c, indicating the MnO_2_ shell was successfully grown. The dynamic diameter of mZGS@Mn was increased to 130.1 ± 14.6 nm (Figure S1, Supporting Information).Figure 1d shows the high resolution TEM (HRTEM) image of mZGS@Mn. Through calculation, the interplanar spacing of 3.13 nm corresponded to the (2,2,0) plane of the cubic spinel. As shown in Figure 1e, all the elements were homogeneously distributed, validating the successful synthesis of mZGS@Mn. The energy dispersive spectrometer (EDS) spectrum demonstrates that mZGS@Mn was composed of O, Ga, Si, Sn, Cr, Mn, Zn, Y (Figure S2, Supporting Information). The X‐ray photoelectron spectroscopy spectra (XPS) validated that mZGS@Mn consists of O, Ga, Si, Sn, Cr, Mn, Zn. Furthermore, the oxidation state of Mn was determined to be +4 due to the Mn 2p1/2 peak and Mn 2p3/2 peak centered at 653.6 and 642.0 eV, respectively with a spin‐energy separation of 11.6 eV, which corresponded with MnO_2_ (Figure S3, Supporting Information). The specific content of Zn, Ga, Sn, Si, Mn was analyzed to be 15.17%, 17.58%, 6.52%, 25.51%, and 4.05% (wt%), respectively (Figure S4, Supporting Information). As depicted in Figure 1f, X‐ray diffraction (XRD) pattern of both mZGS and mZGS@Mn matched well with the standard line of ZnGa2O4 (PDF#38‐1240), indicating ZGS was prepared. In comparison with mZGS, the XRD pattern of mZGS@Mn shows no distinct diffraction peaks, illustrating the amorphous structure of MnO_2_ shell. Besides, the diffraction peaks at small angle were observed in the pattern of mZGS indicates the existence of mesopore structure, and were disappeared in the pattern of mZGS@Mn, indicating the mesopore structure was lost. To prove the pore structure of samples, nitrogen absorption and desorption tests were performed. The typical type IV isotherms verify the mesopore structure of mSiO_2_. The large Brunauer–Emmett–Teller (BET) surface area of mSiO_2_ was calculated to be 659.39 m^2^ g^−1^ with high pore volume of 0.94 cm^3^ g^−1^ and average pore size of 5.75 nm, which was ideal to be the template of ZGS (Figure S5, Supporting Information). The BET surface area of mZGS remained 261.12 m^2^ g^−1^ with pore volume of 0.41 cm^3^ g^−1^, which afforded space for MnO_2_ to grow. The BET surface area of mZGS@Mn was reduced to 72.65 m^2^ g^−1^ with pore volume of 0.106 cm^3^ g^−1^, validating the mesopore was almost filled by MnO_2_. The Fourier transform infrared (FTIR) spectra, suggesting the successive connection of mZGS@Mn with poly(allylamine hydrochloride) (PAH) and polyacrylic acid (PAA) (Figure S7, Supporting Information). The zeta potential of samples at each step significantly changed, proving that eventually AMD3100 molecule was successfully conjugated with mZGS@Mn. The loading content of AMD3100 was calculated to be 4.9% (wt%) with loading efficiency of 51.6% by using high performance liquid chromatography (HPLC). Besides, the dynamic diameter of samples at each step gradually increased from 130.1 ± 14.6 nm of mZGS@Mn to 172.7 ± 8.4 nm of mZGS@Mn‐AMD (Figure S1, Supporting Information).

### 2.2 PersL Performance of mZGS@Mn‐AMD

The PersL spectra of samples located at 600–800 nm and peaked at 702 nm in NIR region (Figure 1g). The PersL of mZGS@Mn was significantly quenched on account of the broad absorption of MnO_2_ (Figure S8, Supporting Information). After the degradation of MnO_2_, PersL (purple line) greatly recovered and increased to ≈5.1 times of that before degradation (blue line). In addition, the PersL performance of a series of samples with different amount of metal ions were studied (Figure S9, Supporting Information). With the increase of metal ions content, the mesopore structure gradually disappeared, and collapsed when the amount of metal ions increased to 1.5 mmol (Figure S10, Supporting Information). Therefore, with the increase of metal ions, MnO_2_ layer, grown on mesopore surface, gradually became thinner. As shown in Figure 1h, the mass fraction of Mn element in mZGS@Mn, calculated by inductively coupled plasma optical emission spectrometer (ICP‐OES), gradually decreased from 12.76% to 0.15%. When the amount of metal ions was 0.5 mmol, the multiples of PersL enhancement was only 1.57, because the amount of MnO_2_ was too much to be completely degraded. The PersL enhancement increased as the MnO_2_ content decreased. Subsequently, the multiples of PersL enhancement decreased, because the MnO_2_ content was too low to quench the PersL. As a result, the optimal amount of metal ions was 1 mmol with 5.1 multiples PersL enhancement. As displayed in Figure 1i, the PersL decay spectrum illustrates the good NIR PersL performance of mZGS in 2 h. The re‐excitation PersL spectra kept consistent, validating the excellent optical stability of mZGS (Figure S11, Supporting Information). The PersL decay images exhibited in Figure 1j indicates that intense PL emission of mZGS in 30 min. The PersL images of PBS containing mZGS@Mn with or without glutathione (GSH) were shown in Figure 1k. When added 10 mm GSH, the PersL dramatically enhanced in 30 min, while the PersL intensity did not change obviously in the group without GSH. The process of MnO_2_ degradation displayed in Figure S12, suggesting that both endogenous GSH and H_2_O_2_ accelerated degradation. Further, the degradation profile of MnO_2_ over time was obtained by ICP‐OES, demonstrating that when encountered H_2_O_2_ and GSH, MnO_2_ significantly decomposed within 10 min, and was almost decomposed in 30 min (Figure 1l).

### 2.3 Specific Targeting Property and Biosafety of mZGS@Mn‐AMD

**Figure 2:**
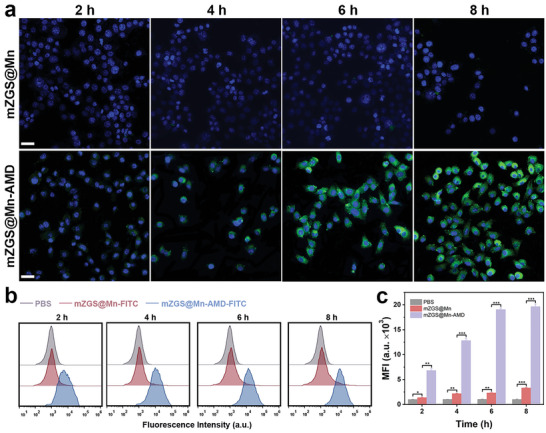
In vitro specific targeting property of the nanoprobe. a) Confocal laser scanning microscopy images of 4T1 cells incubated with mZGS@Mn or mZGS@Mn‐AMD for 2, 4, 6, 8 h, respectively. b) Flow cytometry analysis of 4T1 cells incubated with PBS, mZGS@Mn‐FITC or mZGS@Mn‐AMD‐FITC for 2, 4, 6, 8 h, respectively. c) Mean fluorescence intensity analyzed from (b). *p < 0.05, **p < 0.01, ***p < 0.001, ****p < 0.0001, *n* = 3. Scale bar 50 µm.

In order to investigate the targeting ability of the nanoprobe, 4T1 cells with high level of CXCR4 expression were incubated with the nanoprobe for various time.^[^ ^16^ ^]^ The intracellular faint fluorescence suggested that a small number of nanoprobes were taken up within 2 h (**Figure** **2**a). With the extension of incubation time, the amount of uptake gradually increased and reached the peak at 6–8 h. The negligible fluorescence was observed in mZGS@Mn without AMD3100 conjugation, verifying that mZGS@Mn‐AMD uptake in 4T1 cells was mediated by the interaction between CXCR4 and AMD3100. The results of flow cytometry assays shown in Figures 2b and 2c were consistent with those described above, validating the outstanding targeting ability of the nanoprobe. For further in vivo application, the biosafety of the nanoprobe have to be verified. Normal breast epithelial cell line MCF‐10A and mouse embryonic fibroblast cell line NIH‐3T3 were selected to estimate the cytotoxicity of the nanoprobes. The cell viabilities approached to 100% even if the concentration of the nanoprobes increased to 200 µg mL^−1^, demonstrating the low cytotoxicity of the nanoprobes (Figure S13, Supporting Information). On the basis of the result of hemolysis assays, the red blood cells mostly kept intact even at high concentration of 200 µg mL^−1^ with less than 5% hemolysis ratio, verifying the low blood toxicity (Figure S14, Supporting Information). A week after tail intravenously injection of mZGS@Mn‐AMD, the blood samples were collected from posterior orbital vein of the mice for blood routine tests. No obvious abnormality was found in the mZGS@Mn‐AMD group, proving the biosafety of the nanoprobe (Figure S15, Supporting Information). Moreover, two weeks after tail intravenous injection with the nanoprobes, the main organs of mice were excised for pathologic analysis. No obvious tissue damage was observed after injection with nanoprobe according to hematoxylin & eosin (H&E) staining, further indicating the good biocompatibility (Figure 16, Supporting Information).

### 2.4 Ultrasensitive PersL Image

**Figure 3:**
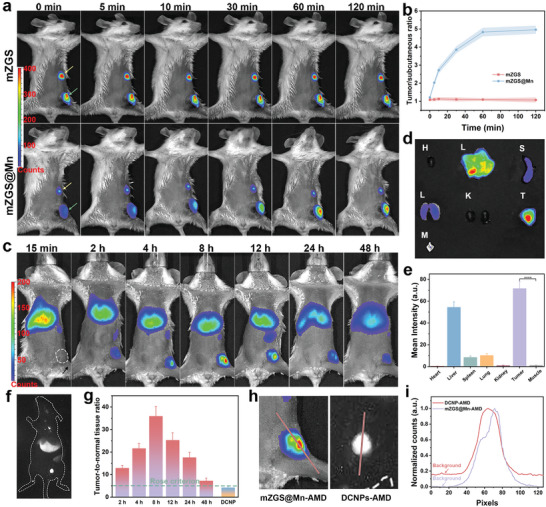
Ultrasensitive PersL image by the nanoprobe. a) PersL images of mice subjected both subcutaneous injection and intratumoral injection with mZGS and mZGS@Mn. The yellow arrows point to subcutaneous injection site and the green arrows point to intratumoral injection site. b) Intratumoral signal‐to‐subcutaneous signal ratio analyzed from (a). c) PersL images of mice intravenously injected with mZGS@Mn‐AMD. d) PersL images and e) mean intensity of excised organs, tumor, and muscle (muscle was obtained from where the arrow points in (c)). f) NIR‐II fluorescent image of mouse intravenously injected with DCNPs‐AMD. g) T/NT signal ratios at different time analyzed from (c) and (f). h) Location of line profile. i) Line profile of photon counts. ****p < 0.0001, *n* = 3.

To test the higher sensitivity of the nanoprobe compared with that of mZGS, equal amounts of samples were injected subcutaneously and intratumorally into the mice. As shown in **Figure** **3**a, the PersL signal intensity of subcutaneous and intratumoral maintained in mZGS group, while gradually enhanced intratumoral signal was observed within 2 h in group mZGS@Mn. The PersL signal intensity was calculated in Figure S17, Supporting Information, from which the trend of signal strength over time is observed more intuitively. Through calculation, the intratumoral/subcutaneous signal ratio rapidly rose within 60 min and reached to at 120 min in mZGS@Mn group, while that fluctuated around 1 in mZGS group (Figure 3b). Therefore, based on the result above, it is easy to draw the conclusion that the degradation of MnO_2_ recovered the PersL, resulting in higher sensitivity. At first, the ultrasensitive nanoprobe was applied in orthotopic breast cancer. 15 min after the intravenous injection of nanoprobe, negligible signal was observed at tumor site. Since 2 h post injection, strong NIR PersL at tumor site was observed, peaked at 8 h post injection and remained considerable intensity 48 h after injection with negligible background interference (Figure 3c). Notably, attributed to the ultrahigh sensitivity, the tumor tissues could be clearly distinguished from surrounding normal tissue since 2 h post injection, which was essential for the subsequent resection. From the ex vivo image shown in Figure 3d, the strongest PersL signal of tumor was observed, compared with that of other organs, illustrating the specific targeting of the nanoprobe. The mean PersL intensity of tumor was 60 times higher than that of surrounding muscles, indicating ultrahigh sensitivity of the nanoprobe. To investigate the pharmacokinetics of mZGS@Mn‐AMD, the Ga concentration in blood of mice was measured (Figure S19, Supporting Information). The Ga concentration in blood versus time curve showed the half‐life of the nanoprobe was 85.4 min. And the biodistribution of mZGS@Mn‐AMD was analyzed by ICP‐MS, indicating the considerable targeting ability(Figure S18, Supporting Information). As a contrast, NaErF_4_@NaYF_4_ (DCNPs), which has been extensive studied, was conjugated with AMD3100 and used for NIR II (≈1550 nm) imaging (Figure 3f).^[^ ^17^ ^]^The T/NT signal ratios at different time point post injection with mZGS@Mn‐AMD were calculated, shown in Figure 3g. The T/NT signal ratio of mZGS@Mn‐AMD rapidly reached 12.9 at 2 h, rose to the peak of 35.9 at 8 h, and remained 7.26 at 48 h, which was far higher than that of DCNPs‐AMD. Moreover, the T/NT signal ratio of the nanoprobe was well above the rose criterion, which supposes that distinguishing image features with 100% certainty requires a T/NT ratio of 5.^[^ ^18^ ^]^ Therefore, the superhigh T/NT ratio allowed tumor readily to distinguish. In order to observe the T/NT ratios of mZGS@Mn‐AMD and DCNPs‐AMD more intuitively, the signal intensity was analyzed according to the profile line drawn in Figure 3h. The background of mZGS@Mn‐AMD was extremely close to 0, while that of DCNPs‐AMD was ≈0.16, explaining the relatively high sensitivity of the nanoprobes (Figure 3i). Hence, the nanoprobe showed distinct superiority over DCNPs for in vivo imaging.

### 2.5 PersL Image‐Guided Orthotopic 4T1 Tumor Resection

**Figure 4:**
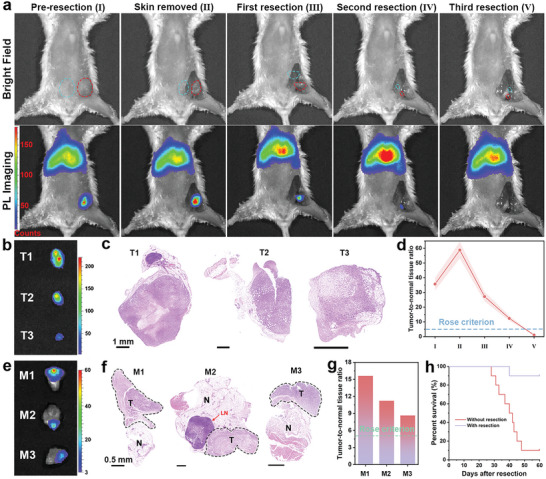
PersL image‐guided surgically resected orthotopic 4T1 tumor after intravenously injection of mZGS@Mn‐AMD. a) PersL images during resection (in the bright field image, red circle and blue circle represent the position of tumor and muscle chosen for quantification, respectively). b) Ex vivo excised tumor imaging. c) H&E staining images of tumor. d) T/NT signal ratios analyzed from (a) (*n* = 3). e) PersL images of three surgical margin (M1, M2, M3). f) H&E staining images of tumor margin. T, N, LN represent tumor, normal tissue, and lymph node, respectively. g) T/NT signal ratios of tumor margin. h) Kaplan–Meier survival rate curve of 10 mice with or without tumor resection.

The strategy of image‐guided tumor resection was depicted in Scheme 1. All PersL images was obtained by excitation with a red LED light for 20 s, followed by immediately exposure for 1 min under imaging system. This process takes the least time that has been reported so far, contributing to convenient and fast intraoperative imaging. At first, preoperative imaging was carried out to judge the tumor location. After first resection, PersL imaging is repeated to determine the tumor residues, navigating the next accurate resection. Until all tumor tissues are removed, evidenced by no PersL signal, the wound is sutured. Surgical resection of orthotopic breast tumor was performed at 8 h postejection of the nanoprobe. The surgical procedure was divided into 5 steps: pre‐resection, skin removed, first resection, second resection, third resection. As shown in **Figure** **4**a, PersL imaging was performed at each step. Compared with pre‐resection, the brighter PersL was observed after skin removed. Smaller and weaker signal was collected after first resection, depicting the position, shape, and size of remnant tumor. With the guidance, second resection was performed, followed by imaging again. The tiny PersL signal demonstrated that micro tumor tissue was left and needed for another resection. After third resection, no signal was observed at original position, suggesting that the tumor was completely removed. From bright field images, the residual tumor tissues were hard to distinguish after second resection, but it became feasible attributed to the ultrasensitive PersL imaging. The luminance differences of excised tumor tissues at each step were in agreement with that of intraoperative imaging (Figure 4b). The H&E staining demonstrated that the diseased tumor tissue adhered to a small part of the normal tissues (Figure 4c). Therefore, accurate and complete surgical removal was accomplished. The T/NT signal ratios of intraoperative imaging were calculated and shown in Figure 4d, while the PersL intensity at tumor site and surrounding muscle was shown in Figure S20, Supporting Information. An extraordinarily high T/NT ratio of 58.8 was obtained during surgery. Unexpectedly, for the <2 mm size of tumor tissues, although the PersL was relatively weak, the T/NT ratio still reached up to 12.4, considerably higher than rose criterion.

The T/NT ratio was nearly 1 after third resection, proving that there is no residual tumor tissue. The same procedure of tumor resection with imaging guidance was performed on two other mice (Figures S21 and S22, Supporting Information). To further demonstrate that tumor tissues could be distinguished from normal tissues, three pieces tumor margin attached with normal tissue were excised for imaging. As shown in Figure 4e, PersL signal was only observed in tumor tissues. In addition, in the H&E staining images, the shapes of tumor tissues corresponded to that of the luminous spot in PersL images (Figure 4f). The T/NT ratios were calculated to be 15.6, 11.2, and 8.65 for M1, M2, and M3, respectively, and both were higher than rose criterion (Figure 1g). Consequently, tumor tissues were readily distinguished from surrounding healthy tissues with the ultrasensitive imaging. With resection surgery, the survival rate was significantly improved compared to control group, as shown in Figure 4h.

### 2.6 PersL Image‐Guided Multiple Microtumor Resection

**Figure 5:**
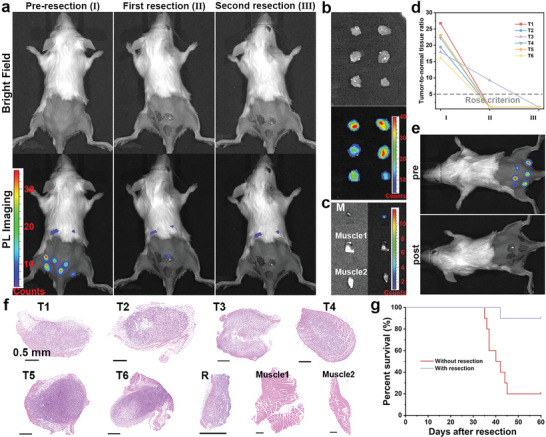
PersL image‐guided surgically resected multiple‐microtumor after intravenously injection of mZGS@Mn‐AMD. a) PersL images during resection. b) Ex vivo PersL image of resected microtumors. c) PersL image of residual tumor tissue and muscles. d) T/NT signal ratios analyzed from (a). e) Bioluminescence images before and after resection. f) H&E staining images of microtumors, tumor residue, and muscles. T1–T6 represent six microtumor, R represents remnant of T4. g) Kaplan–Meier survival rate curve of 10 mice with or without tumor resection.

Encouraged by the results of orthotopic breast cancer model, the nanoprobe was then applied in multiple‐micro tumor model to demonstrate if the nanoprobe could recognize tiny tumor. As The multiple‐microtumor model was constructed by subcutaneously planting a small number of 4T1 cells in multiple places. As shown in **Figure** **5**a, six micro lesions were clearly observed without background interference from preoperative imaging, which were highly consistent with the bioluminescence (Figure 5e). Among 21 tiny tumors, all were identified by PersL imaging, verifying the accuracy of identifying small tumors (Figures S23 and S24, Supporting Information). After first resection, a small PersL signal was still observed, indicating the residual tumor tissue. The surgical removal was achieved due to no signal after second resection. The ex vivo imaging as shown in Figurer 5b indicated the harvested tumors posed relative high luminance. The tumor residue was significantly brighter than surrounding muscles, proving the ultrasensitive imaging (Figure 5c). For multiple microtumor, the T/NT signal ratios in preoperative imaging ranged from 16.1 to 26.8, considerably higher than rose criterion (Figure 5d). Even for residual tumor tissue, the T/NT ratio remained 9.24, adequate to distinguish from the surrounding normal tissues. All the T/NT ratios decreased to about 1, demonstrating no tumor residue was left. Meanwhile, the postoperative bioluminescence imaging confirmed all tumor was removed (Figure 5e). H&E staining images illustrates that all the entire borderline of microtumors with size of about 2 mm except T4, were effectively distinguished from normal tissue by ultrasensitive imaging (Figure 5f). Furthermore, the remnants of T4 (<1 mm) could be distinguished from surrounding healthy tissue as well. The survival rate of mice subjected image‐guided surgery was significantly improved within 60 days (Figure 5g).

### 2.7 PersL Image‐Guided Liver Tumor Resection

**Figure 6:**
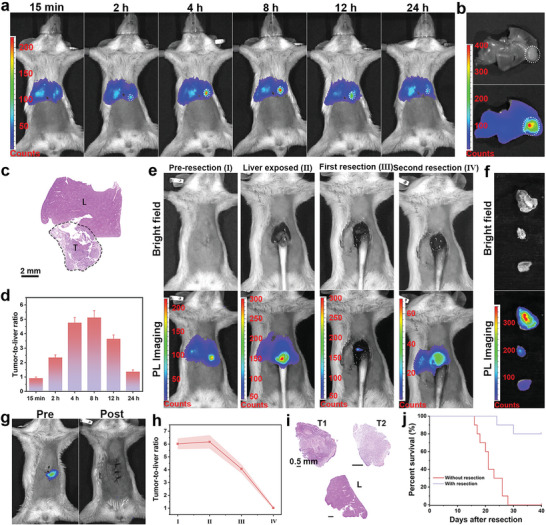
PersL image‐guided surgically resected liver tumor after intravenously injection of mZGS@Mn‐AMD. a) PersL images of mouse with intravenous injection of nanoprobe at different time. b) Ex vivo PersL image of harvested liver. c) H&E staining images of liver. T and L represent tumor tissue and healthy liver tissue, respectively. d) T/L signal ratios analyzed from (a). e) PersL images during resection. f) Ex vivo tumor imaging. g) Bioluminescence images of mouse before and after resection. h) T/L signal ratios in intraoperative imaging. i) H&E staining images of tumor, tumor residue, and liver tissue. j) Kaplan–Meier survival rate curve of 10 mice with or without tumor resection. *n* = 3.

Nanoparticles are usually enriched in liver, hindering hepatoma imaging. Therefore, liver tumor model was built to investigate whether the nanoprobe could identify 4T1 liver‐implanted tumor. At first, the nanoprobe retention time at tumor site was studied. Within 15 minutes of the intravenous injection, the signal was uniformly distributed in liver (**Figure** **6**a). At 2 h post injection, brighter portion was observed compared with surrounding liver tissue at left lobe of liver. The signal at tumor site gradually got stronger and achieved maximum intensity at 8 h post injection. A considerable strong intensity was remained after 12 h injection and weakened at 24 h post injection. The ex vivo imaging of excised liver was performed, from which the significantly stronger signal was observed at tumor site compared with healthy liver. The H&E staining demonstrated the brighter part was lesion as shown in Figure 6c. Thus, relying on the ultrasensitive imaging, the tumor tissue could be distinguished from surrounding liver tissue. The tumor‐to‐liver (T/L) signal ratios were analyzed in Figure 6d. Within first 2 h, the T/L signal ratio slightly increased from 1 to 2.3, subsequently dramatically increased to 5.2 at 8 h post injection. The T/L ratio remained 3.6 at 12 h after injection and decreased to 1.4 at 24 h. The T/L ratio kept relatively high for a considerable period of time, available for image‐guided 4T1 liver‐implanted tumor resection. Consequently, liver tumor resection was then performed with the guidance of PersL imaging. According to preoperative image, the position of tumor was roughly determined (Figure 6e). Based on this, after laparotomy, the pathological liver was exposed. In intraoperative image, the tumor was much brighter than surrounding liver tissues, and the tumor margins were clearly delineated, in agreement with the bioluminescence image (Figure 6g). After first resection, there were a small and weak signal remained, suggesting the residual tumor tissue. After another resection, the PersL signal was uniformly distributed and there were no obvious bright spots, demonstrating tumor was completely resected. The excised tumor was obviously brighter than normal liver tissue, even for the tiny tumor residue (Figure 6f). The tumor was completely removed and without residue in body, evidenced by the bioluminescence imaging (Figure 6g). As shown in Figure 6h, the preoperative T/L signal ratio was 6.0 and increased to 6.2 after liver exposure, feasible to distinguish the tumor from healthy tissues. Further, the T/L signal ratio of residual tumor (<2 mm) kept 4.1, allowing to identify. After second resection, the T/L signal ratio sharply drop to about 1, indicating no tumor residue. The H&E staining images proved that the excised part was diseased tissue (Figure 6i). In addition, another two mice were subjected to surgical removal and were shown (Figures S25, S26, Supporting Information).The survival rate of mice with image‐guided tumor resection was extremely improved, while all mice in control group dead within 30 days (Figure 6j).

### 2.8 PersL Image‐Guided Lung Metastasis Resection

**Figure 7:**
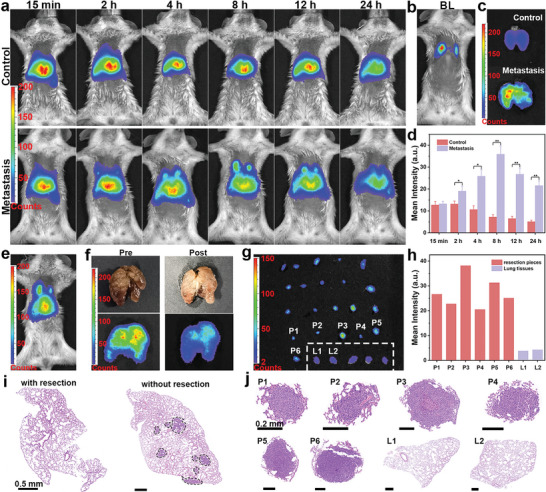
PersL image‐guided surgically resected lung metastasis after intravenously injection of mZGS@Mn‐AMD. a) PersL images of mouse with intravenous injection of nanoprobe at different time (the control group was healthy mice). b) Bioluminescence image, c) ex vivo PersL image of harvested lung. d) Mean PersL intensity at lung analyzed from (a). e) PersL images before surgery. f) Ex vivo lung images before and after resection. g) PersL images of resected pieces and normal lung tissues (normal lung tissues were in the dashed box). h) Mean PersL intensity of resected pieces and normal lung tissues. i) H&E staining images of lung with and without resection. j) H&E staining images of resected pieces and normal lung tissues. *n* = 3.

Furthermore, metastatic lung cancer model was constructed to study the nanoprobe applied for metastasis. The retention time of nanoprobe was first investigated, as shown in **Figure** **7**a. At 2 h post injection, the weak signal at lung was observed. Since 4 h, the signal at lung in experimental group was significantly stronger than that in control group, validating that the specific targeting of the nanoprobe for metastatic tumor. The signal intensity at lung gradually increased and achieved maximum at 8 h after injection. Besides, the signal at lung was consistent with the bioluminescence shown in Figure 7b. The ex vivo images of lungs in Figure 7c proving that pathological lung was remarkably brighter than the healthy lung. The mean PersL intensity of lung at different time after injection was analyzed in Figure 7d. At 15 min, there is no significant difference between experimental group and control group. The intensity remarkably increased in experimental group within 8 h, while the intensity slowly decreased in control group. The significant difference between two group was observed since 2 h post injection. Additionally, the mean PersL intensity peaked at 8 h after injection. Thus, the image‐guided surgical removal began 8 h after injection. At first, the intraoperative imaging was carried out, as shown in Figure 7e. Surgical resection was performed ex vivo for the different experimental conditions. The ex vivo images of lung before and after resection and corresponding photographs were shown in Figure 7f. Many micro lesions were observed pre‐operation and were excised after resection according to photographs. In comparison with after resection, the obviously stronger signal was observed before resection. Despite their small size, the excised lesions were brighter than lung tissues, as shown in Figure 7g. 5 resected pieces and 2 pieces of normal lung tissues was randomly selected for mean intensity analysis. The mean PersL intensity of resected pieces was approximately 5.1–7.5 times that of healthy lung tissues (Figure 7h). Therefore, the metastasis was feasible to distinguish from normal tissue attributed to the ultrahigh T/NT signal ratio. To prove if the metastasis was completely removed, lung tissue with and without resection were stained by H&E. As shown in Figure 7i, no diseased region was observed in the image with resection, while 8 tiny metastatic lesions were observed in the image without resection. Besides, the selected pieces (P1–P6) were all metastatic lesion, evidence by H&E staining images. Thus, rely on image‐guided resection, metastasis was accurately recognized and was thoroughly removed. Besides, the surgical procedure of another two mice were shown (Figures S27, S28, Supporting Information).

## 3 Conclusion

In summary, based on the property of PLNPs without background fluorescence interference, an ultrasensitive nanoprobe was constructed for image‐guided tumor resection. The MnO_2_ shell quenched PersL in normal tissue and was degraded in TME, resulting in that the sensitivity of tumor imaging was about 4.8 times that pure PLNPs without MnO_2_ shell. Notably, the T/NT signal ratio of the nanoprobe was 9 times that of DCNP (NIRIIb imaging), demonstrating the ultrahigh sensitivity. Various tumor models were built to investigate whether the nanoprobe could determine residual tumor tissue. The intraoperative T/NT signal ratio reached up to 58.8 for intact primary 4T1 tumor and remained 12.4 for residual tumor (<2 mm). For multiple‐microtumor, all small tumors were identified, as well as the tumor residue. As for 4T1 liver‐implanted tumor, the residual tumor tissue was still successfully recognized rely on PersL imaging. Due to the ultrasensitive imaging, tumor tissue was clearly distinguished from surrounding normal tissue. In addition, the nanoprobe is expected to be used for the identification and resection of metastasis. Remarkably, the entire PersL imaging process took less than 1.5 min, convenient for image‐guided surgery. Therefore, depending on the ultrasensitive imaging method, we envision future utility in image‐guided tumor resection in different conditions.

## 4 Experimental Section

### Synthesis of mSiO_2_

First, triethanolamine (TEA, 0.18 g) was dissolved in DI water (36 mL). Then, hexadecyl trimethyl ammonium chloride (CTAC) aqueous solution (25%, 24 mL) was added, and the mixture was stirred under 60 °C for 1 h. Subsequently, 4 mL of tetraethyl orthosilicate (TEOS) was dissolved in cyclohexane (16 mL) and was dropwise added in the mixture with gently stirred for 18 h. Afterward, the mSiO_2_ was obtained by centrifugation and washed with ethyl alcohol and water. The CTAC template was removed by calcining under 550 °C for 5 h.

### Synthesis of mZGS

Zn_1.3_Ga_1.4_Sn_0.3_O_4_:Cr_0.005_, Y_0.003_ was synthesized by template method. Briefly, metal ions were prepared into aqueous solution according to the chemical formula and then added into mSiO_2_ (100 mg) with homogeneous mixing. Finally, the mixture was heat to 60 °C under vacuum overnight and calcined under 900 °C for 3 h.

### Synthesis of mZGS@Mn

First, mZGS (15 mg) was dispersed in DI water (40 mL). Then, KMnO_4_ aqueous solution (5 mg mL^−1^, 12 mL) was added in the mixture under stirred. After 10 min, formamide (1.2 mL) was added and the mixture was allowed to react under ultrasound for 30 min. The mZGS@Mn was collected by centrifugation and washed with water.

### Surface Modification of mZGS@Mn

First, mZGS@Mn (20 mg) was added in poly(allylamine hydrochloride) (PAH) aqueous solution (10 mg mL^−1^, 50 mL) and was allowed to react under ultrasound for 2 h. The solution was centrifuged and washed with water for three times. Then, the samples were dispersed in polyacrylic acid (PAA aqueous) solution (10 mg mL^−1^, 50 mL) under ultrasound. After 2 h, the samples were collected by centrifugation and washed with water for three times. AMD 3100 (1 mg) was conjugated with the samples by amidation to obtain final mZGS@Mn‐AMD. In brief, mZGS@Mn (10 mg) was dispersed in 10 mL of MES buffer. EDC (2 mg) was added under stirring, followed by adding 4 mg NHS. After 1 h, the pH of the mixture was adjusted to 7.2, and AMD3100 (1 mg) dissolved in ethanol was added. After 12 h, mZGS@Mn‐AMD was washed with water and collected by centrifugation. In addition, after reaction, the supernatant was collected to quantify of AMD3100 by using HPLC with mobile phase consisting of 0.1% trifluoroacetic acid (212 nm).

### Synthesis and Modification of DCNPs

DCNPs were synthesized according to previous study.^[^ ^19^ ^]^ The obtained DCNPs were conjugated with PAA by ligand exchange.^[^ ^20^ ^]^ AMD3100 were then conjugated with the PAA‐modified DCNPs by amidation, the same as mZGS@Mn. In brief, DCNPs‐PAA (10 mg) was dispersed in 10 mL of MES buffer. EDC (2 mg) was added under stirring, followed by adding 4 mg NHS. After 1 h, the pH of the mixture was adjusted to 7.2, and AMD3100 (1 mg) dissolved in ethanol was added. After 12 h, DCNPs‐AMD was washed with water and collected by centrifugation.

### In Vitro Degradation

In vitro degradation of mZGS@Mn was tested by quantifying the accumulated Mn content by ICP‐OES. Briefly, the samples (0.3 mg mL^−1^) were dispersed in different solution (PBS, PBS with 0.01 m GSH, PBS with 0.01 m GSH and 5 × 10^−5^ m H_2_O_2_) at 37 °C. At given time intervals, a part of supernatant was collected for ICP‐OES measurements.

### Cellular Uptake Assay

First, the samples (mZGS@Mn and mZGS@Mn‐AMD, 2 mg mL^−1^) were stirred with FITC (0.05 mg mL^−1^) overnight to obtain mZGS@Mn‐FITC and mZGS@Mn‐AMD‐FITC for the assay. 4T1 cells (5 × 10^4^ cells per well) were seeded in 24‐well plate at 5% CO_2_ and 37 °C overnight. Then, the mZGS@Mn‐FITC and mZGS@Mn‐AMD‐FITC (50 µg mL^−1^) were added and incubated for 2, 4, 6, 8 h, respectively, followed by washing with PBS for five times. For obtaining CLSM images, the cells were fixed with paraformaldehyde solution (4%) for 20 min, and incubated with DAPI for 15 min. For flow cytometry assay, cells were digested, washed with PBS for two times, and resuspended in PBS.

### In Vitro Cytotoxicity

Normal breast epithelial cell line MCF‐10A and mouse embryonic fibroblast cell line NIH‐3T3 were subjected to standard cck‐8 assays. MCF‐10A cells and NIH‐3T3 cells were seeded in 96‐well plates with density of 5 × 10^3^ cells per well and were further cultured for 12 h at 5% CO_2_ and 37 °C. Then the cells were incubated with different dose of mZGS@Mn‐AMD for 24 h. Subsequently, the culture medium was replaced by fresh medium containing 10% cck‐8. After 1 h incubation, the cells were analyzed by microplate reader.

### Pharmacokinetics and Biodistribution of mZGS@Mn‐AMD

After intravenously injection of mZGS@Mn‐AMD (200 µL, 2 mg mL^−1^), blood samples were obtained from the posterior orbital vein of the mice at specific time point post injection. Then the SiO_2_ was removed by stirred with NaOH (2 m) for 24 h, and then excessive aqua regia solution was added to dissolve other components. As for biodistribution in vivo of mZGS@Mn‐AMD, the harvested organs and tumors were dissolved in aqua regia solution and heated to remove the acid, followed by treatment by NaOH solution and aqua regia solution in sequence. Ga concentration in blood, organs, and tumors was measured by ICP‐MS.

### Animal Tumor Models

All animal experiments were approved by the Animal. Ethics Committee of Xiamen University (No. XMULAC20180037). All animal models were using 6 weeks female Balb/c mice, purchased from GemPharmatech LLC. Orthotopic breast cancer model: 4T1‐luc cells (1 × 10^6^) were suspended in 100 µL PBS and injected into fat pad of mice. The inguinal mammary gland near the nipple was selected for tumor cell inoculation. PL imaging was carried out after the tumor size reaching ≈300 mm^3^. Multiple‐microtumor model. 4T1‐luc cells (1 × 10^5^) were suspended in 20 µL and randomly subcutaneously injected in the back. After 3 days, imaging was carried out. Lung metastatic model: 4T1‐luc cells (2 × 10^5^) were suspended in 200 µL and tail intravenous injected into mice. After two weeks, imaging was carried out. 4T1 liver‐implanted tumor model: 4T1‐Luc cells were suspended in 1 mL of serum‐free 1640 medium. Add 1 mL Matrigel to adjust the cell concentration to 1 × 10^5^ per 20 µL. Mice were anesthetized and performed a midline abdominal incision. 20 µL 4T1 cell/Matrigel solution was slowly injected in the bottom of the left lobe of liver. Finally, the bleeding was stopped and the abdominal wall was closed. PL imaging was performed after a week.

### Persistent Luminescence Imaging

Unless otherwise specified, 0.4 mg nanoprobes in 200 uL PBS buffer was tail intravenous injected into mice. At given time intervals, mice were irradiated by 659‐nm light‐emitting diode (LED) at 40 mW cm^−2^ for 20 s and then rapidly placed into IVIS Lumina II imaging system (Caliper Life Science, Inc., USA) with 60 s exposure. All images were processed by Living Image (Caliper Life Science, Inc., Version 4.3.1).

### Statistical Analysis

Data were collected from at least three independent measurements (*n* ≥ 3). All the data are presented as mean ± standard deviation (SD) unless otherwise indicated. Means were compared using a two‐sided Student's *t*‐test, *p* < 0.05, *p* < 0.01, *p* < 0.001, and *p* < 0.0001 were considered to be statistically significant with showing by *, **, ***, and ****, respectively. Survival rate was evaluated using Kaplan–Meier analysis. All the experiments were performed in triplicate to ensure experimental reproducibility. Graphs were drawn by using Origin 2021 (Version 9.8.0.200, OriginLab Corp.).

## Conflict of Interest

The authors declare no conflict of interest.

## Supporting information

Supporting Information

## Data Availability

The data that support the findings of this study are available in the supplementary material of this article.
