# Supplementary material for: Near‐Infrared Persistent Luminescence Nanoprobe for Ultrasensitive Image‐Guided Tumor Resection
Source: Adv Sci (Weinh). 2023 Apr 23;10(18):2207486. doi: 10.1002/advs.202207486 (PMC10288272; doi:10.1002/advs.202207486)
Supplement: Supplementary file 1 — Supporting Information [file ADVS-10-2207486-s001.pdf]

Supporting Information

Near-Infrared Persistent Luminescence Nanoprobe for Ultrasensitive Image-guided Tumor Resection

Peng Lin, Junpeng Shi, Ye Lin, Qian Zhang, Kexin Yu, Lin Liu, Liang Song, Yile Kang, Maochun Hong*, Yun Zhang**

Materials: All chemical reagents were used without further purification. Hexadecyl trimethyl ammonium chloride (CTAC, 97%), Triethanolamine (TEA, 99%), Zinc acetate dihydrate ($Zn(OAc)_2·2H_2O$, 99.99%), Gallium nitrate hydrate ($Ga(NO_3)_3·xH_2O$, 99.99%), Chromic acetate ($Cr(OAc)_3$, 99.9%), Yttrium nitrate hexahydrate ($Y(NO_3)_3·6H_2O$, 99.99%), Stannic chloride hydrated ($SnCl_4·5H_2O$, 99.995%), Formamide (99.5%), Glutathione (Reduced, 98%) were purchased from Aladdin-Reagent Co. Ltd (China). Cyclohexane and Potassium permanganate (KMnO4) were acquired from Sinopharm Chemical Reagent Co., Ltd (China). Tetraethyl orthosilicate (TEOS), Poly(allylamine hydrochloride) (PAH, Mw = 17500), Polyacrylic acid (PAA, Mw = 2000) were purchased from Sigma–Aldrich (USA). AMD 3100 was purchased from MedChemExpress (USA).

Characterization: The transmission election microscopy (TEM) images were collected by Hitachi H-7650 (Hitachi Co., Ltd., Japan) with 100 kV accelerating voltage. High resolution transmission election microscopy (HRTEM) and elements mapping images were obtained by Talos F200X unit (Thermo Fisher Co., Ltd., USA) with 200 kV accelerating voltage. X-ray diffraction patterns were acquired by Miniflex 600 (Rigaku Co., Ltd., Japan). Zeta potential and hydrodynamic diameter of samples were measured by Brookhaven Omni (Nano Brook Omni, Brookhaven Instruments Corporation, USA). Autosorb-IQ (Quantachrome Instruments, USA) was used to obtain N_2 adsorption–desorption isotherms and pore-size distributions. Ultraviolet–visible-near-infrared light absorption spectra were acquired from Agilent Cary 5000 UV–Vis-NIR spectrophotometer (Agilent Technologies Co., Ltd.,

USA). Mn element contents were measured by ULTIMA 2 inductively coupled plasma optical emission spectrometer (ICP-OES, HORIBA Scientific Co., Ltd.,French). Persistent luminescence spectra were collected by FluoroMax-4 (Horiba Instruments Inc, USA). Persistent luminescence images were acquired by Tanon-4600SF (Tanon, China).

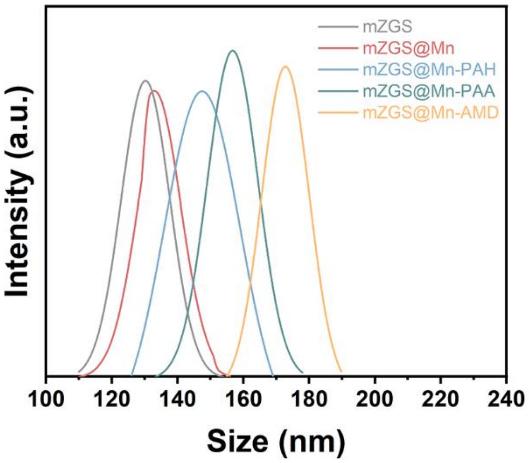

Figure S1. Hydrodynamic diameter of samples.

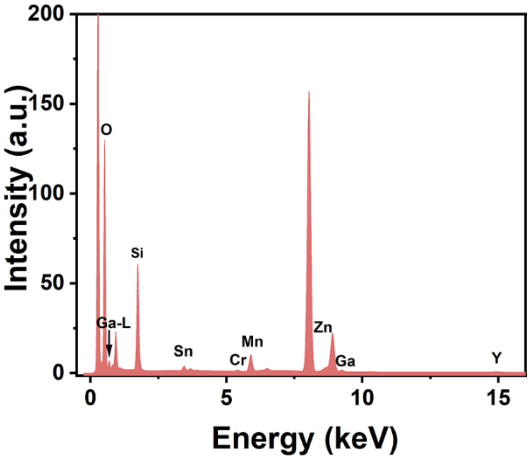

Figure S2. EDS spectrum of mZGS@Mn.

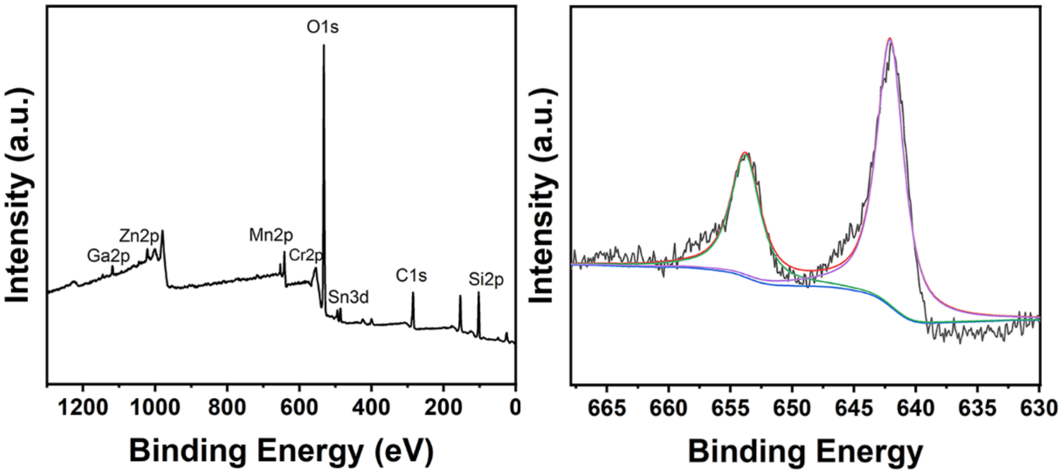

Figure S3. XPS spectra of mZGS@Mn.

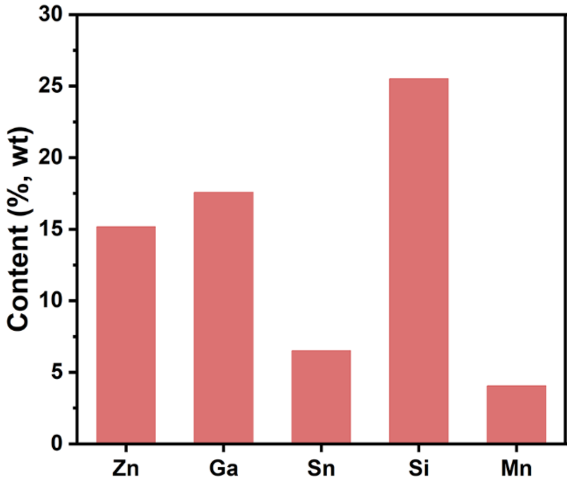

Figure S4. Element content (wt%) in mZGS@Mn measured by ICP-OES

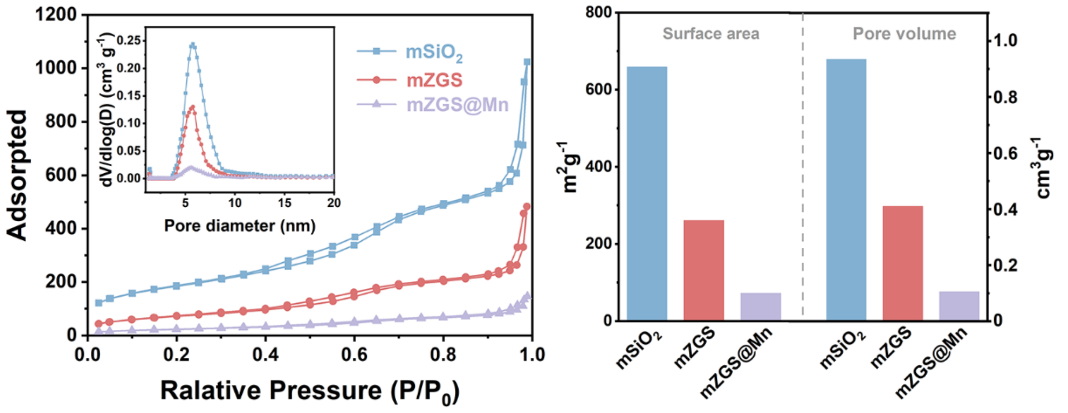

Figure S5. (left) N_2 absorption–desorption isotherms (inset is corresponding pore-size distribution) of mSiO2, mZGS and mZGS@Mn; (right) BET surface area and pore volume of samples.

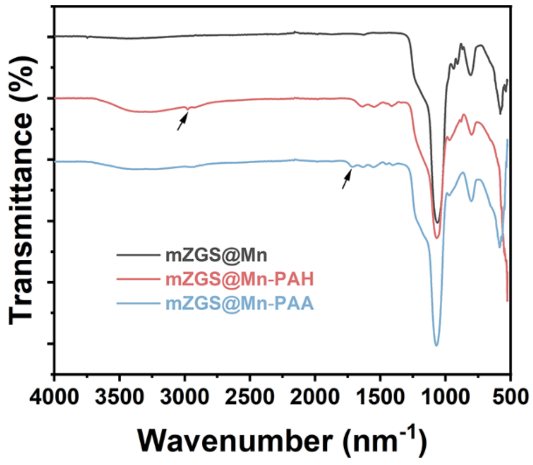

Figure S6. FTIR spectra of mZGS@Mn, mZGS@Mn-PAH and mZGS@Mn-PAA.

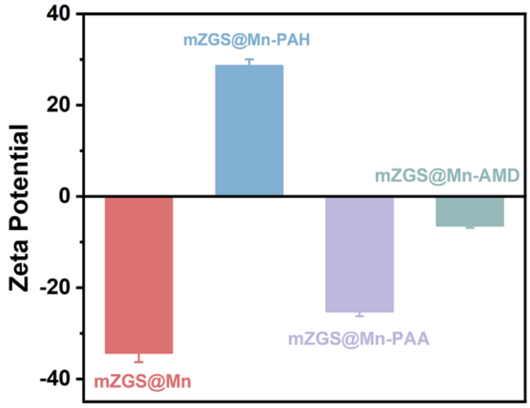

Figure S7. Zeta potential of mZGS@Mn, mZGS@Mn-PAH, mZGS@Mn-PAA and mZGS@Mn-AMD. n = 3.

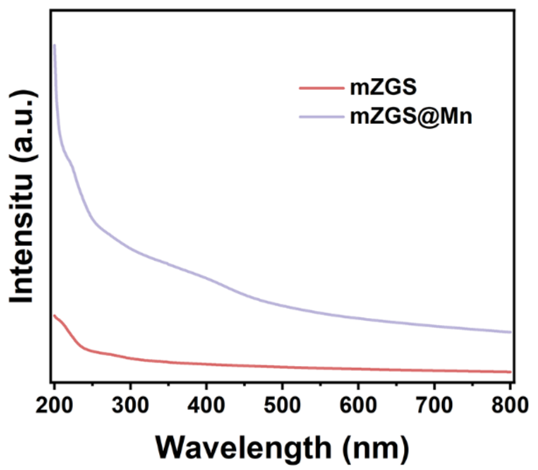

Figure S8. Uv-visible-near infrared absorption spectra of mZGS and mZGS@Mn.

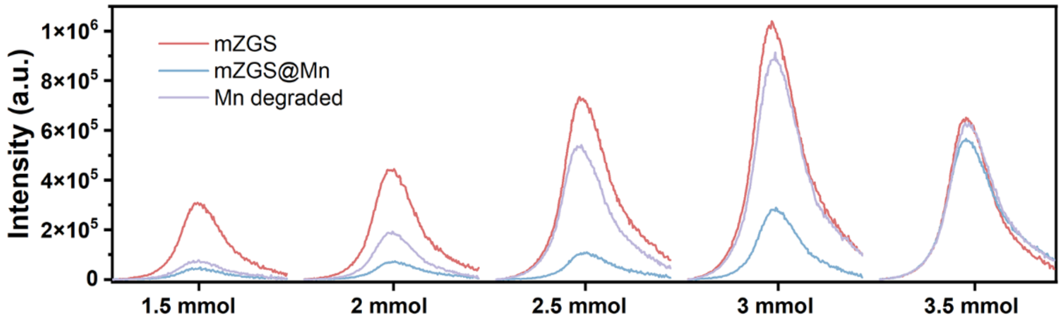

Figure S9. PersL spectra of samples with various addition amount of metal ions.

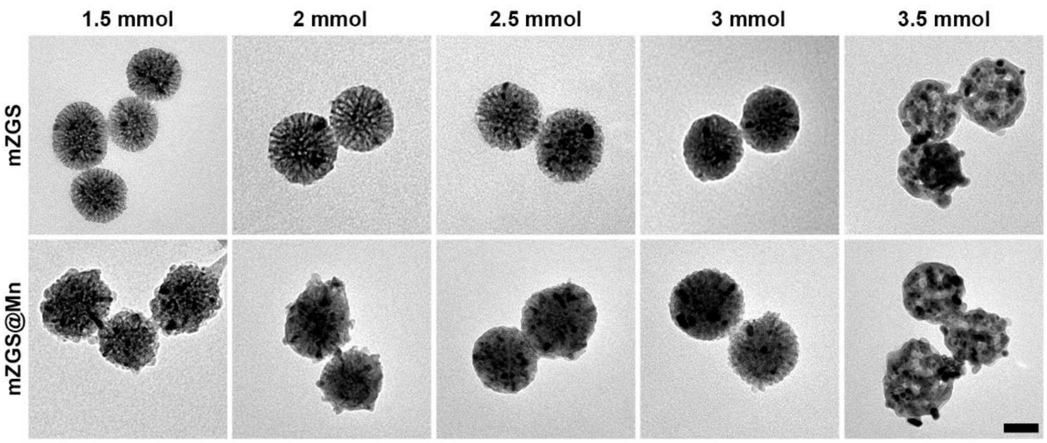

Figure S10. TEM images of mZGS and mZGS@Mn with various addition amount of metal ions.

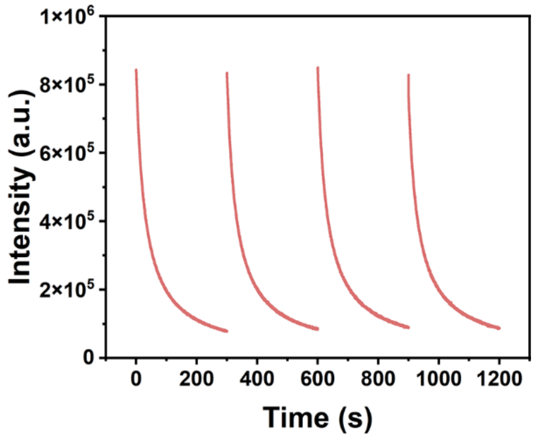

Figure S11. Re-excitation PersL decay curve of mZGS.

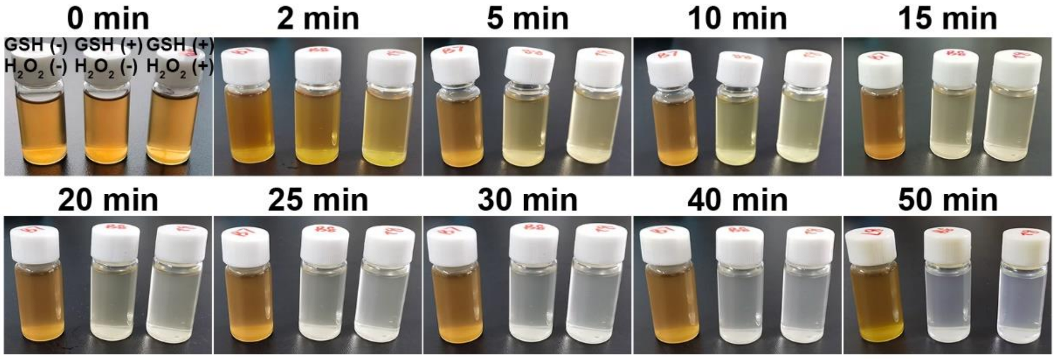

Figure S12. Photographs during MnO_2 degradation test.

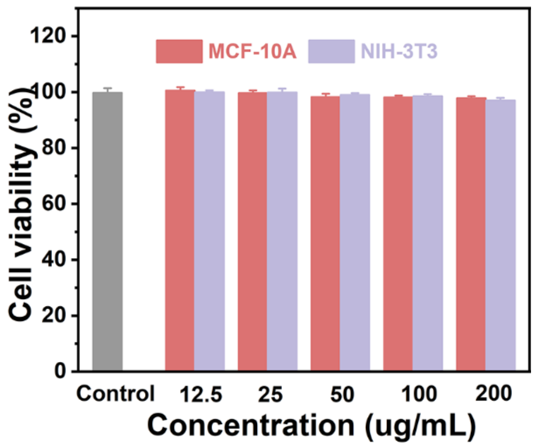

Figure S13. Cell viability of MCF-10A and NIH-3T3 treated with mZGS@Mn-AMD for 24 h. n = 3.

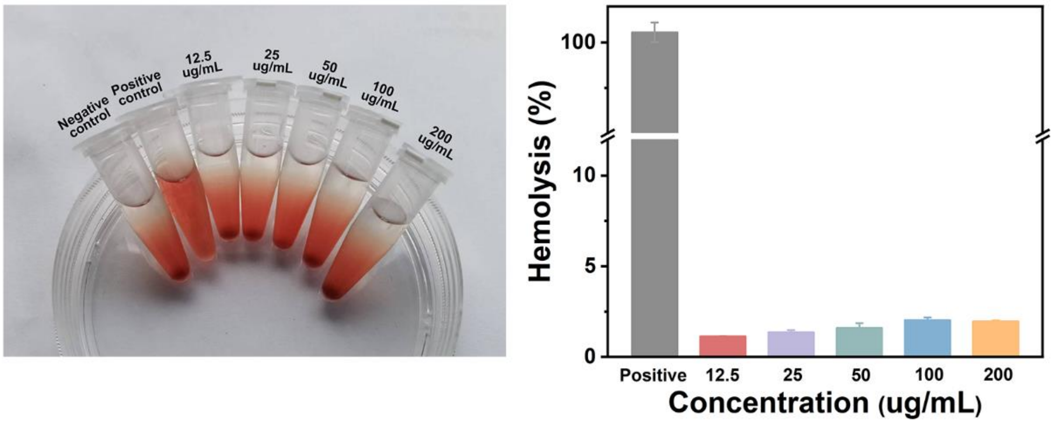

Figure S14. Hemolysis rates of red blood cell treated with mZGS@Mn-AMD for 3 h. n = 3.

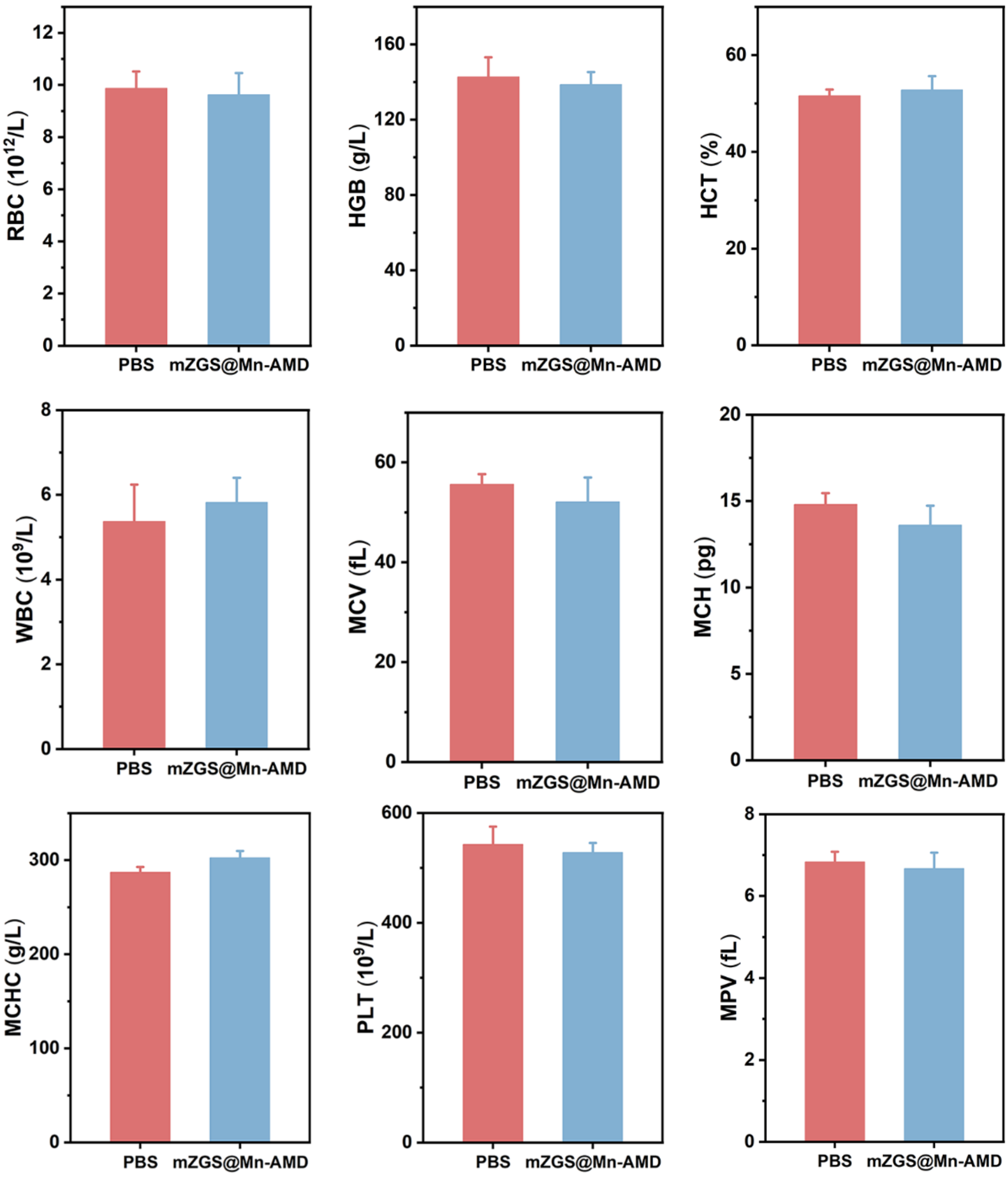

Figure S15. The blood routine examination of mice 7 days after receiving intraveneously injection of mZGS@Mn-AMD. RBC: red blood cells, HGB: hemoglobin, HCT:hematocrit, WBC: white blood cells, MCV: mean corpuscular volume, MCH: mean corpuscular hemoglobin, MCHC: mean corpuscular hemoglobin concentration, PLT: platelet, MPV: mean platelet volume. n = 3.

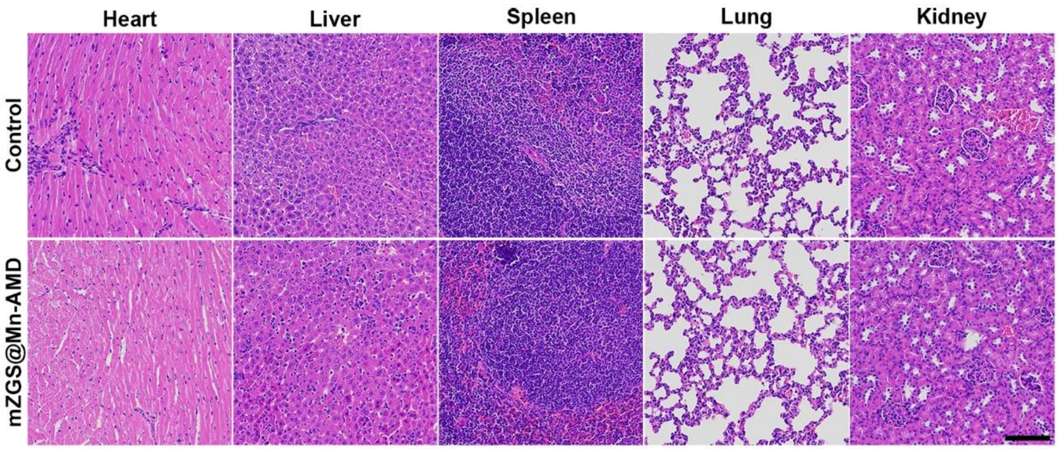

Figure S16. H&E staining images of primary organs of mice after 2 weeks intravenous injection with PBS and mZGS@Ms-AMD.

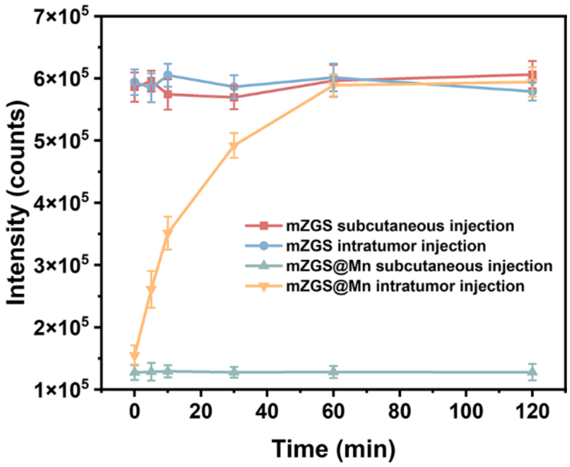

Figure S17. PersL intensity caculated from the imaging of mice subjected both subcutaneous injection and intratumoral injection with mZGS and mZGS@Mn. n = 3.

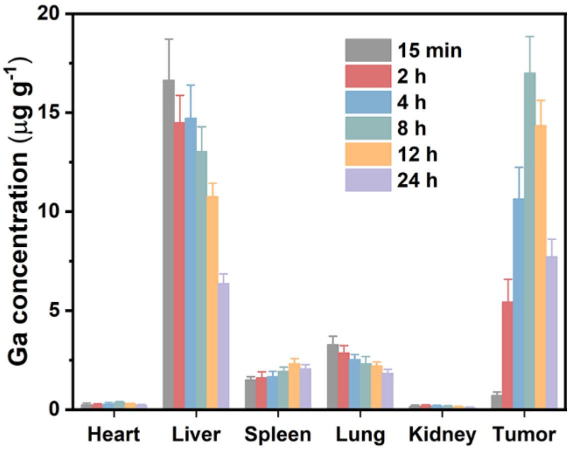

Figure S18. Biodistribution of mZGS@Mn-AMD in orthotopic tumor-bearing mice for 24 h measured by ICP-MS. n = 3.

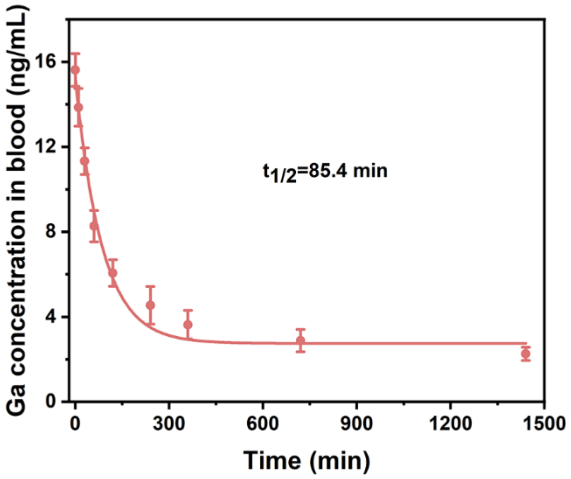

Figure S19. Blood concentration versus time curve of mZGS@Mn-AMD nanoprobe in mice. n = 3.

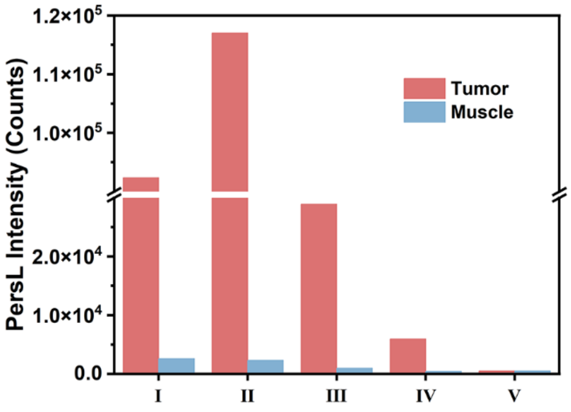

Figure S20. PersL intensity of tumor and surrouding muscle (the position for quantification was circled in Figure 4a bright field image).

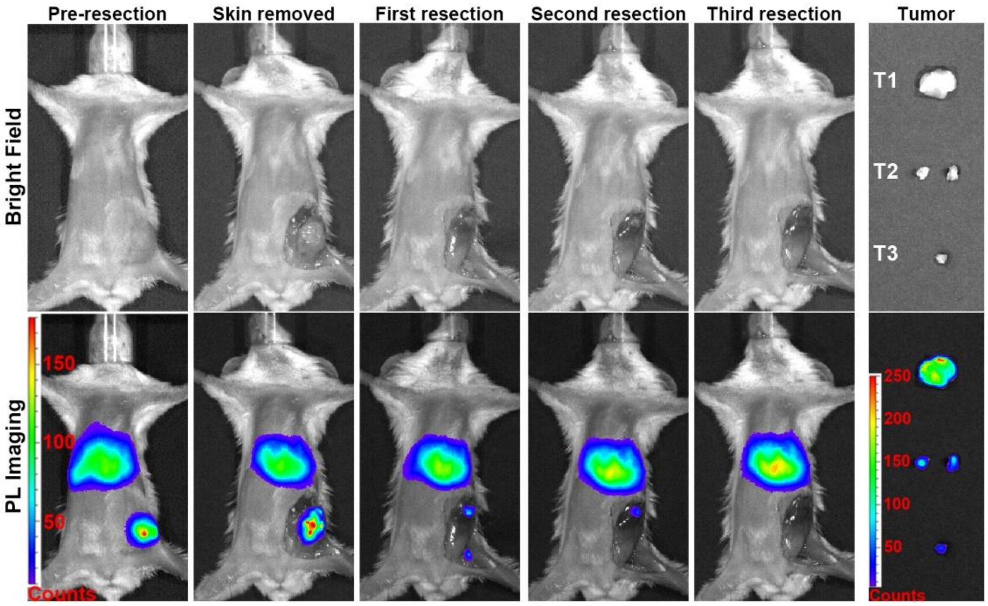

Figure S21. PersL images of mouse 2 during primary 4T1 tumor resection.

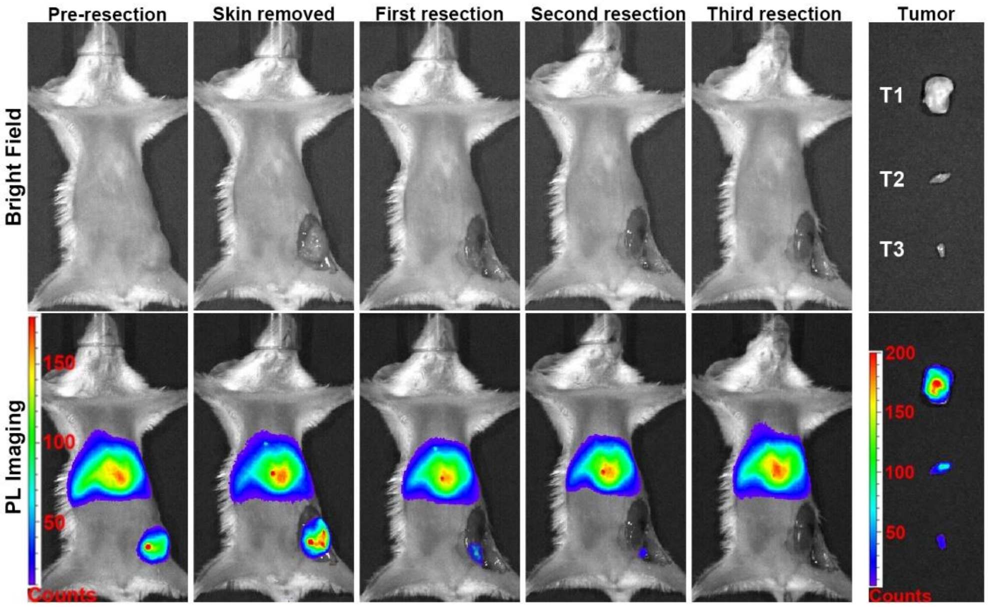

Figure S22. PersL images of mouse 3 during primary 4T1 tumor resection.

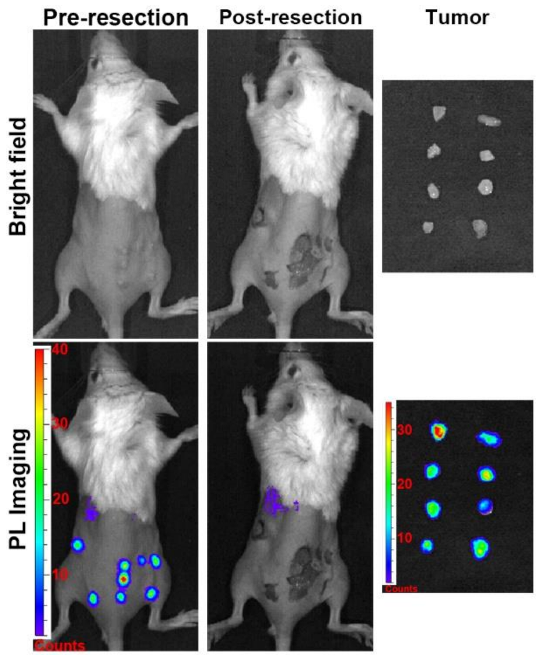

Figure S23. PersL images of mouse 2 during multiple-microtumor resection.

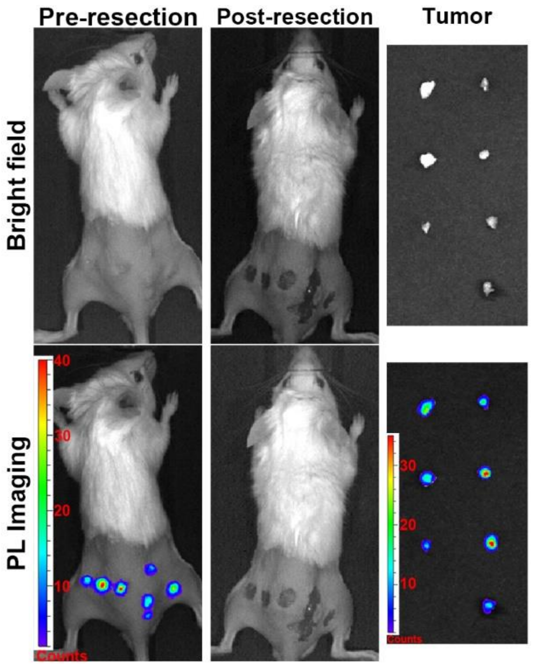

Figure S24. PersL images of mouse 3 during multiple-microtumor resection.

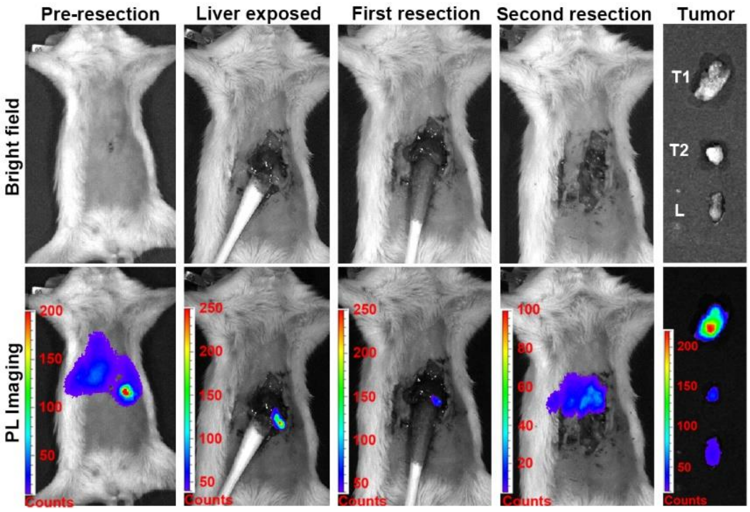

Figure S25. PersL images of mouse 2 during liver tumor resection.

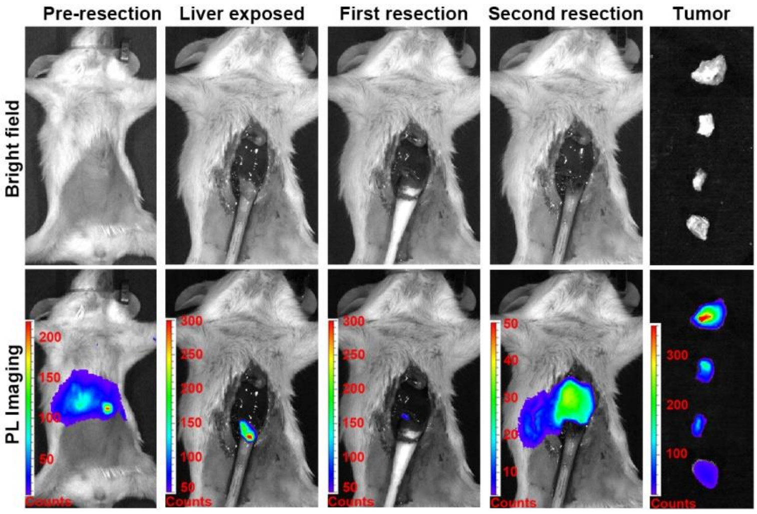

Figure S26. PersL images of mouse 3 during liver tumor resection.

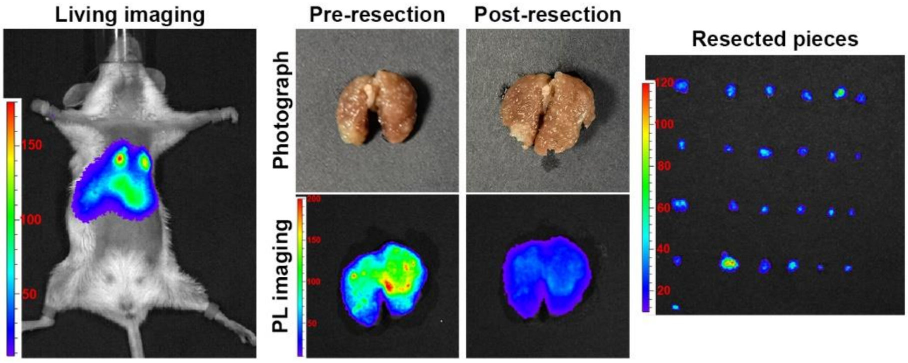

Figure S27. PersL images of mouse 2 during lung metastasis resection.

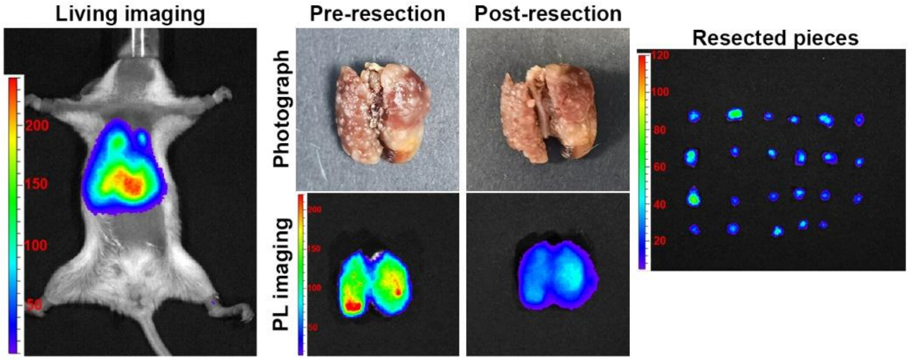

Figure S28. PersL images of mouse 3 during lung metastasis resection.
